# Graph-based deep learning for drug-drug interaction prediction: a systematic review

**DOI:** 10.3389/fchem.2026.1806549

**Published:** 2026-05-11

**Authors:** Xiaoqing Liu, Xue Yu, Qi Dai

**Affiliations:** 1 College of Sciences, Hangzhou Dianzi University, Hangzhou, China; 2 College of Life Sciences, Zhejiang Sci-Tech University, Hangzhou, China

**Keywords:** drug-drug interaction, graph attention networks, graph contrastive learning, graph convolutional networks, prediction methods

## Abstract

Identifying potential drug–drug interactions (DDIs) is crucial for drug development and therapeutic safety, motivating increasing efforts in computational DDI prediction. Although several surveys have summarized recent advances, a systematic review that explicitly organizes existing studies from the perspective of graph-based deep learning paradigms is still lacking. In this work, we present a comprehensive review of graph-based methods for DDI prediction, with a particular focus on three representative technical routes: graph convolutional networks (GCNs), graph attention networks (GATs), and graph contrastive learning (GCL). We review and categorize representative DDI prediction models according to these three paradigms, highlighting their modeling strategies, advantages, and limitations in capturing molecular structures, heterogeneous interactions, and robust representations. We then discuss key challenges and future research directions, emphasizing multi-modal data integration and model interpretability. This review aims to provide a structured overview of the current landscape and to serve as a practical reference for the development of more accurate, robust, and interpretable DDI prediction models.

## 1 Introduction

Drug–drug interactions (DDIs) refer to the pharmacological or toxicological effects that arise when two or more drugs are administered concurrently, potentially leading to enhanced or diminished therapeutic efficacy or even unexpected adverse reactions. Such interactions may alter treatment outcomes and, in severe cases, induce significant safety issues, including increased toxicity or therapeutic failure (Levy and Ragueneau-Majlessi, 2019). With the growing adoption of personalized medicine and multi-drug therapy in clinical practice, the prediction and assessment of DDIs have attracted widespread attention.

Traditional DDI prediction strategies typically rely on experimental validation or rule-based inference grounded in known interaction patterns. However, these approaches exhibit substantial limitations. First, experimental assays are costly, time-consuming, and difficult to scale alongside rapidly expanding drug spaces. Second, rule-based inference depends heavily on existing DDI knowledge, making it incapable of discovering novel or previously unreported interactions, thus restricting prediction coverage. Moreover, such methods often lack mechanistic understanding of drug structures and biological activities, resulting in suboptimal ability to reveal underlying DDI patterns. These drawbacks underscore the urgent need for efficient and accurate computational frameworks to improve DDI discovery.

The rapid growth of biomedical data, together with advances in machine learning, deep learning, and network modeling, has brought new opportunities for computational DDI prediction. In particular, graph neural networks (GNNs)—a class of deep learning frameworks designed for graph-structured data—have demonstrated remarkable success in modeling complex drug interaction networks. In DDI graphs, nodes typically represent drugs and edges encode pharmacological associations or interaction events. Through iterative graph convolution or message passing, GNNs capture structural dependencies and biochemical relationships between drugs and their molecular targets, thereby enhancing predictive accuracy (Lin et al., 2023).

Building upon GNNs, graph contrastive learning (GCL) has recently emerged as a powerful self-supervised paradigm for DDI prediction. The core idea of GCL is to construct multiple graph views of drug interaction events, generate positive and negative sample pairs, and optimize embedding representations via contrastive objectives. By encouraging similar drug pairs to remain close in the embedding space while pushing dissimilar ones apart, GCL enables models to better learn latent interaction patterns, especially under data-scarce scenarios, making it well-suited for DDI tasks where annotated samples are limited.

To address these challenges, researchers have proposed a series of DDI models that integrate GNNs with contrastive learning. For example, Wang et al. (Wang et al., 2021) introduced a multi-view graph contrastive representation learning framework to jointly capture molecular structures and pharmacological associations. Jiang et al. (Jiang M. et al., 2024) further developed a relation-aware graph embedding mechanism with collaborative contrastive learning to improve representation learning for multi-relational DDIs. Although such strategies have significantly improved the ability to capture drug interaction patterns, DDI prediction still faces several open challenges. Data sparsity remains a critical bottleneck, particularly for newly approved drugs with limited interaction records, which restricts the generalization ability of predictive models (Ma et al., 2018). In addition, current DDI algorithms are predominantly black-box architectures with limited interpretability, making it difficult for clinicians to understand decision-making processes and apply predictions in medical practice (Chen et al., 2016; Lin et al., 2020; Zitnik et al., 2018).

As accumulating evidence suggests that uncovering potential DDIs is crucial for drug development and therapeutic safety, an increasing number of studies have been dedicated to computational DDI prediction. Although previous surveys have reviewed advances from varying perspectives, a comprehensive analysis focusing on graph-based computational methodologies remains lacking. In this work, we provide a systematic and integrative review of graph-based deep learning approaches for DDI prediction. First, we introduce the foundational concepts of graph-based deep learning techniques. Next, we summarize representative GNN- and GCL-based DDI prediction models. Finally, we discuss current challenges and outline future research directions.

## 2 Graph‐based computational methods for predicting DDIs

Graph Neural Networks (GNNs) are a class of deep learning models designed for graph-structured data, with theoretical foundations rooted in Graph Theory and representation learning. GNNs operate through a message passing mechanism, where node features are iteratively aggregated and updated from their neighbors, enabling the learning of node-, edge-, or graph-level embeddings. Representative models such as Graph Convolutional Network and Graph Attention Network leverage convolutional operations and attention mechanisms, respectively, to effectively capture complex topological dependencies. Building upon this, Graph Contrastive Learning (GCL) introduces a self-supervised paradigm that further enhances representation learning in GNNs. By generating multiple augmented views of the original graph, GCL performs contrastive learning in the embedding space. Its core idea is to maximize the agreement between positive pairs (different augmented views of the same node or graph) while minimizing the agreement between negative pairs, thereby pulling positive samples closer and pushing negative samples apart.

Despite the high accuracy of traditional clinical experimental analysis methods in drug–drug interaction (DDI) prediction, their widespread application is severely limited by disadvantages such as being time-consuming, labor-intensive, and costly. In recent years, automated computational approaches, including machine learning and deep learning techniques, have been widely adopted for DDI prediction. Conventional machine learning–based DDI prediction models predominantly utilize chemical fingerprints and similarity-based features as inputs. Chemical fingerprints, which are sequence-based representations, are capable of characterizing specific properties of drugs, such as chemical substructures, associated targets, and side effects. More recently, an increasing number of deep learning–based techniques have been applied to DDI prediction tasks, as they can automatically learn more expressive feature representations from data, thereby significantly improving the performance of DDI prediction models.

### 2.1 GNN‐based computational methods for predicting DDIs

Over the past years, several GNN-based DDI prediction frameworks have been proposed, which can be broadly categorized into GCN-based models and GAT/attention-based models (Table 1). These methods vary in their treatment of molecular graphs, integration of multi-modal information, attention mechanisms, and interpretability.

**TABLE 1:** Summary of existing Graph-based methods for DDI prediction in terms of mechanisms, techniques, and task types.

Method	Mechanism	Techniques	Ref
DPDDI	GCN	Structural GCN + pairwise prediction	Feng et al. (2020)
GCN-BMP	GCN	Patch-based GCN	Chen et al. (2020)
MR-GNN	GCN	Multi-resolution + dual graph	Xu et al. (2019)
BDN-DDI	GCN	Bilinear dual-view	Ning et al. (2023)
DSN-DDI	GCN	Dual-view + generalization	Lin et al. (2023)
MDNN	GCN	Multi-modal fusion	Lyu et al. (2021)
Kang et al.	GCN	Feature + topology fusion	Kang et al. (2022)
MK-GCN	GCN	Multi-kernel convolution	Wang et al. (2022a)
Multi-Kernel GNN	GCN	Adaptive kernel integration	Zhao et al. (2025)
Sparse Hypergraph	GCN	High-order hypergraph	Nguyen et al. (2022)
BM GNN	GCN	Baseline molecular GNN	Abbas et al. (2025)
AutoDDI	GCN	Neural architecture search	Gao et al. (2024)
SSI-DDI	GAT	Substructure attention	Nyamabo et al. (2021)
DeepDDS	GAT	Attention-based synergy prediction	Wang et al. (2022b)
Size-adaptive GNN	GAT	Motif-level explainable attention	Nyamabo et al. (2022)
Substructure GNN	GAT	Motif-level interpretability	Yang et al. (2022)
MathEagle	GAT	Heterogeneous graph + multi-head	Hou et al. (2024)
DrugDAGT	GAT	Dual-attention + contrastive learning	Chen et al. (2024)
DA-GAT	GAT	Domain-adaptive + interpretable attention	Zhang et al. (2025)
MASMDDI	GAT	Soft-mask message passing	Lin et al. (2024)
GRACE	GCL	Graph encoder and contrastive loss	Zhu et al. (2020)
EGCL	GCL	Augmentation-based contrastive learning	Zhu et al. (2021)
GraphCL	GCL	Node/Edge augmentation, contrastive loss	You et al. (2020)
AD-GCL	GCL	Graph adversarial augmentation, contrastive loss	(Suresh et al.)
BGRL	GCL	Bootstrapped target representation, contrastive alignment	Thakoor et al. (2021)
Barlow Twins	GCL	Cross-correlation loss, self-supervised	Zbontar et al. (2021)
VICReg	GCL	Variance-invariance-covariance regularization	Bardes et al. (2021)
MRGCDDI	GCL	Multi-relational graph encoder, contrastive loss, no augmentation	Li et al. (2026)
CLDDI	GCL	Contrastive pretraining + dual-view fusion	Xu et al. (2023)
Deep GCL	GCL	Graph encoder and contrastive pretraining	Jiang et al. (2024b)
SimGRACE	GCL	Graph encoder, contrastive loss, no augmentation	Xia et al. (2022)
MPHGCL-DDI	GCL	Meta-path based heterogeneous GNN, contrastive loss	Hu et al. (2024)
GCL-DDI	GCL	Molecular graph, drug similarity, dual-view contrastive alignment	Ding et al. (2025)
MMFF-DDI	GCL	Molecular graph and side-effects, contrastive alignment	Zhong et al. (2025)
MOLGAECL	GCL	Pretraining via graph autoencoder, contrastive fine-tuning	Li et al. (2025)
MSHGCL	GCL	Hierarchical graph aggregation, multi-scale contrastive loss	Ge et al. (2025)
DDVR	GCL	Drug visual embedding, contrastive loss	Xie et al. (2025)

#### 2.1.1 GCN-based methods

**FIGURE 1:**
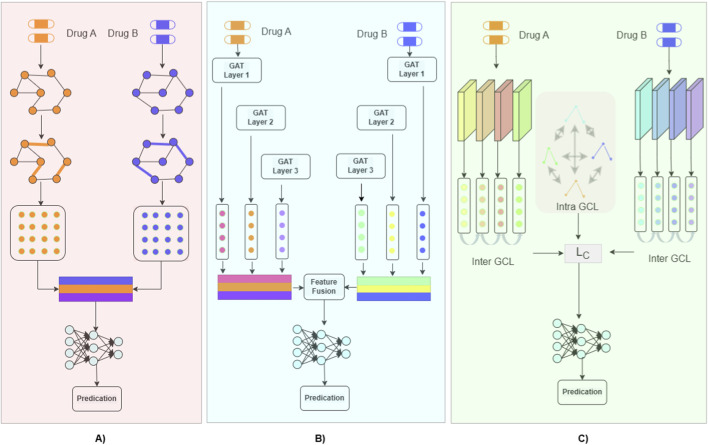
Overview of graph-based computational methods for drug–drug interaction prediction. **(A)** Overview of GCN-based computational methods for DDI prediction. Construction of molecular graphs and corresponding node and edge features based on the chemical structures of the two drugs, graph Convolutional Network (GCN) models are applied to separately learn feature representations (embeddings) from each drug molecular graph, and the learned drug representations are fused and fed into a multilayer perceptron (MLP) to perform binary or multi-class DDI prediction. **(B)** Overview of GAT-based computational methods for DDI prediction. The molecular structures of two input drugs are first provided as input, and Graph Attention Networks (GATs) are employed to learn hierarchical feature representations from each drug molecular graph. Through multi-head attention mechanisms, informative features are selectively aggregated and fused. The fused representations are then fed into a multilayer perceptron (MLP) to perform binary or multi-class DDI prediction. **(C)** Overview of GCL-based computational methods for DDI prediction. The molecular structures of two input drugs are first provided as input. An inter-molecular graph contrastive learning (Inter-GCL, refers to the relationships, differences, or interactions between the ganglion cell layer and adjacent retinal layers) module is employed to capture feature representations at different structural levels across drug molecular graphs, while an intra-molecular graph contrastive learning (Intra-GCL, refers to features, measurements, or structural changes occurring within the ganglion cell layer itself) module is used to learn representations of fine-grained structural units within each drug molecule. The learned representations are further integrated through multi-head attention mechanisms. The fused features are then fed into a multilayer perceptron (MLP) to perform binary and multi-class DDI prediction.

Graph Convolutional Networks (GCNs) constitute the foundational approach for learning structural representations of drugs. GCN-based DDI models typically treat each drug as a molecular graph, applying message passing to aggregate features from neighboring atoms (Figure 1A). Early works such as DPDDI (Feng et al., 2020) and GCN-BMP (Chen et al., 2020) demonstrated that GCNs alone could achieve competitive binary classification performance by learning atom-level features and pairwise interactions without requiring external biological annotations.

To enhance structural expressiveness, several models introduced multi-resolution and multi-view aggregation. MR-GNN (Xu et al., 2019) adopted hierarchical aggregation and dual-graph representation, while BDN-DDI (Ning et al., 2023) and DSN-DDI (Lin et al., 2023) implemented bilinear dual-view representation learning for more generalized interaction modeling. Subsequently, MGDDI (Geng et al., 2024) extended this concept by modeling multi-scale graph features, capturing both fine-grained and coarse-grained molecular patterns.

Recognizing that DDIs arise from complex biochemical mechanisms, some models incorporated multi-modal information. MDNN (Lyu et al., 2021) and Kang et al. (Kang et al., 2022) integrated molecular graphs with protein targets, side-effect profiles, or topological embeddings to predict multi-class interaction events, demonstrating a shift from binary detection toward clinically interpretable interaction type classification.

To further improve expressive power, kernel-based GCNs and high-order graph structures were introduced. MK-GCN (Wang F. et al., 2022) and Multi-Kernel GNN (Zhao et al., 2025) utilized multiple convolution kernels to capture diverse structural patterns, while Sparse Hypergraph Networks (Nguyen et al., 2022) employed hypergraph modeling to represent high-order polypharmacy relations. Foundational baselines like Abbas et al. (Abbas et al., 2025) reaffirmed the effectiveness of basic molecular GCNs, and AutoDDI (Gao et al., 2024) applied neural architecture search (NAS) to optimize GCN architectures automatically.

Single-view structural encoders evolved into multi-resolution and multi-view models, then into multi-modal fusion and high-order interaction modeling, culminating in automated architecture optimization. GCNs remain a core building block for structurally informed DDI prediction.

#### 2.1.2 GAT/Attention-based methods

While GCNs aggregate neighborhood information uniformly, Graph Attention Networks (GATs) introduce learnable attention weights to emphasize the most relevant substructures and molecular interactions (Figure 1B). This family of models emphasizes interpretability, heterogeneity, and transferable representations.

Early attention-based approaches such as SSI-DDI (Nyamabo et al., 2021) modeled substructure–substructure interactions, assigning attention scores to motifs to improve interpretability. This idea evolved into size-adaptive substructure learning, implemented in DeepDDS (Wang J. et al., 2022), Size-adaptive GNN (Nyamabo et al., 2022), and Substructure-aware GNN (Yang et al., 2022), which allowed motif-level explanations and accommodated heterogeneous functional groups.

Later works extended attention mechanisms to heterogeneous networks. MathEagle (Hou et al., 2024) modeled drugs in heterogeneous attributed graphs, integrating pharmacological and side-effect information with multi-head attention. DrugDAGT (Chen et al., 2024) introduced dual-attention graph transformers augmented with contrastive learning, reflecting the broader adoption of transformer-like architectures in molecular graph modeling.

Attention-based models also addressed domain transfer and generalization. Substructure DA-GAT (Zhang et al., 2025) combined interpretable substructure attention with domain-adaptive learning, enabling robust cross-dataset and cross-domain predictions—a critical requirement for clinical applicability.

These methods progressed from substructure attention for interpretability, through multi-head attention for heterogeneous graph integration, to dual-attention transformers and domain-adaptive architectures. They provide a flexible, interpretable framework aligning with the biological reality that not all molecular substructures contribute equally to DDIs.

### 2.2 GCL‐based computational methods for predicting DDIs

Graph Contrastive Learning (GCL) is a self-supervised learning paradigm that learns node- or graph-level embeddings by constructing augmented views of graph data and performing contrastive learning. The core idea of GCL is to maximize the agreement between positive samples while minimizing the agreement between negative samples, such that positive sample pairs are pulled closer in the embedding space, whereas negative sample pairs are pushed apart. Through this mechanism, the model is able to capture essential structural information from graphs. The general contrastive loss functions are shown in Equation 1 and Equation 2:

$$sim\left(h_{i},h_{j}\right)=\frac{h_{i}^{T}h_{j}}{\left\|h_{i}\left\|\;\right\|h_{j}\right\|}$$ (1)

$$L_{contrast}=-\sum_{\left(i,j\right)\in P}\log\frac{\exp\left(sim\left(h_{i},h_{j}\right)/\tau \right)}{\sum_{k\in N}\exp\left(sim\left(h_{i},h_{k}\right)/\tau \right)}$$ (2)

where $\left(h_{i},h_{j}\right)$ denotes a positive pair (e.g., representations of the same drug under different augmented views), $h_{k}$ denotes a negative sample (e.g., representations of different drugs), P denotes the set of all positive pairs, and N denotes the set of comparison samples including both positive and negative pairs. $sim\left(h_{i},h_{j}\right)$ denotes a similarity measure such as cosine similarity, and τ is a temperature parameter that controls the distribution smoothness.

Graph Contrastive Learning (GCL) has emerged as a promising approach for DDI prediction by combining self-supervised learning with graph representation learning (Table 1). GCL methods learn robust drug embeddings by maximizing the agreement between augmented views of graphs while minimizing redundancy, reducing reliance on labeled datasets and enhancing model generalization (Figure 1C).

Early GCL methods focused on graph-level self-supervised pretraining and augmentation strategies. Deep Graph Contrastive Representation Learning (Zhu et al., 2020) introduced graph-level contrastive pretraining, learning embeddings through a standard graph encoder and a contrastive loss function. Empirical Graph Contrastive Learning (Zhu et al., 2021) empirically evaluated different graph augmentation strategies, showing that appropriate augmentations significantly improve embedding quality. Similarly, Graph Contrastive Learning with Augmentations (You et al., 2020) leveraged node and edge perturbations to generate multiple views for contrastive alignment. To improve robustness, Adversarial Graph Augmentation (Suresh et al., 2021) introduced adversarial perturbations in the contrastive learning pipeline, enhancing model resilience to noisy or incomplete molecular graphs. Bootstrapped Graph Representation Learning (Thakoor et al., 2021) employed a self-supervised bootstrap mechanism, aligning target representations across different views without requiring negative samples. Furthermore, redundancy reduction methods like Barlow Twins (Zbontar et al., 2021) and variance-invariance-covariance regularization in VICReg (Bardes et al., 2021) provided additional self-supervised constraints to stabilize graph embedding learning.

As DDI prediction involves complex molecular interactions, several methods extended GCL to multi-relational and dual-view scenarios. Multi-Relational Graph Contrastive DDI (MRGCDDI) (Li et al., 2026) applied contrastive learning on multi-relational drug graphs, capturing heterogeneous interactions among different drug modalities without requiring augmentation. CLDDI (Xu et al., 2023) and Deep GCL for DDI (Jiang Z. et al., 2024) introduced dual-view contrastive learning, aligning embeddings from molecular graphs and drug similarity networks, enabling robust multi-class DDI event prediction. SimGRACE (Xia et al., 2022) presented a simplified GCL framework, demonstrating that even minimalistic contrastive approaches can achieve effective binary DDI prediction without explicit augmentations.

To better capture structural and relational semantics, several methods adopted heterogeneous graph modeling and hierarchical learning. MPHGCL-DDI (Hu et al., 2024) leveraged meta-path-based heterogeneous GNNs in a contrastive learning framework, encoding relational semantics across drug, target, and side-effect networks. MSHGCL (Ge et al., 2025) introduced multi-scale hierarchical aggregation, combining local substructures with global graph context via contrastive objectives. Dual-view GCL-DDI (Ding et al., 2025) utilized dual-view molecular and similarity graphs to enhance multi-class DDI prediction, aligning embeddings from complementary representations.

Multi-modal GCL approaches integrate diverse drug-related information to further improve predictive performance. MMFF-DDI (Zhong et al., 2025) fused molecular graphs, protein targets, and side-effect information using a contrastive learning objective, achieving robust embeddings for multi-class DDI event prediction. MOLGAECL (Li et al., 2025) combined graph autoencoder pretraining with contrastive fine-tuning, leveraging unsupervised molecular structure learning before DDI prediction. Dual-Drug Visual Representation (Xie et al., 2025) embedded dual-drug visual features and applied contrastive alignment, demonstrating that visual embeddings of molecular structures can complement graph-based features.

## 3 Discussion

In recent years, graph neural networks (GNNs) have attracted increasing attention and experienced rapid development in the field of drug–drug interaction (DDI) prediction. This paper presents a systematic review of graph-learning-based DDI prediction methods, with a particular focus on three representative technical paradigms: graph convolutional networks (GCNs), graph attention networks (GATs), and graph contrastive learning (GCL). Existing studies demonstrate that GCNs are effective in modeling drug molecular structures and drug–drug relational networks, showing strong capability in capturing local topological features. GAT-based approaches, by incorporating attention mechanisms, assign differentiated weights to neighboring nodes and exhibit enhanced expressive power in characterizing heterogeneous interactions and key relational patterns. In contrast, GCL-based methods improve the robustness of representation learning through unsupervised or weakly supervised strategies, demonstrating unique advantages in scenarios with limited labeled data or high levels of noise. Overall, GNNs provide an effective modeling paradigm for integrating structural information and relational semantics, significantly advancing the development of DDI prediction.

Despite the remarkable progress achieved by deep learning-based DDI prediction methods, several critical challenges remain, and future research may focus on further exploration and optimization along the following two directions. First, with respect to multi-modal data integration and utilization, the mechanisms underlying drug–drug interactions involve complex biological processes. These interactions are influenced not only by chemical structures but also by genomic expression profiles, protein–protein interaction networks, metabolic pathways, and real-world medication behaviors. Relying on a single data modality is insufficient to comprehensively characterize the multi-level features of DDIs. Future studies may leverage multi-modal deep learning frameworks, such as multi-modal graph neural networks (MGNNs) or unified representation learning models, to jointly model and reason over heterogeneous data sources, including drug structures, gene expression data, and electronic health records. By constructing a unified embedding space, it becomes possible to capture latent cross-modal associations, thereby improving the accuracy and generalization ability of DDI prediction models. With the continuous expansion of public biomedical databases, this research direction is well supported by both data availability and practical application scenarios.

Second, in terms of enhancing model interpretability, although many deep learning-based DDI prediction models achieve superior predictive performance, their black-box nature limits their adoption in clinical decision-making and drug development contexts. Future research may integrate structural attention mechanisms in GNNs with interpretability methods such as GNNExplainer and PGExplainer to visualize key molecular substructures, important target sites, or potential interaction pathways. In addition, incorporating rule-based reasoning and path mining techniques based on knowledge graphs can help extract biologically grounded explanatory evidence from existing pharmacological knowledge. Such strategies can improve the transparency and credibility of model predictions and facilitate the discovery of underlying interaction mechanisms.

In summary, with the continuous accumulation of multi-modal biomedical data and the ongoing evolution of graph neural networks and deep learning techniques, DDI prediction is moving toward a more systematic, intelligent, and interpretable paradigm. Future studies should aim to enhance the capability of models to integrate complex heterogeneous data while balancing predictive performance and biological interpretability. This will further promote the practical deployment of GNN-based models in real-world clinical medication safety assessment and novel drug development. Through continuous optimization of model architectures, training strategies, and knowledge integration mechanisms, more efficient and reliable DDI prediction systems can be established.
